# Identification of the cation/H+ exchanger genes in peanut and functional analysis of *AhCAX8* in response to salt stress

**DOI:** 10.3389/fpls.2026.1900784

**Published:** 2026-07-16

**Authors:** Fangyan Ma, Hongfeng Wang, Guang Yuan, Chen Zhang, Yang Xu, Dunwei Ci, Guanchu Zhang

**Affiliations:** 1 Shandong Peanut Research Institute, Qingdao, China; 2 Technology Center, China Tobacco Shandong Industry Co., Ltd., Jinan, China; 3 College of Advanced Agricultural Sciences, Zhejiang Agriculture and Forestry (A&F) University, Hangzhou, China

**Keywords:** CAX, drought, expression, peanut, salt

## Abstract

The Cation/H^+^ exchanger (CAX) is an important class of transmembrane transporter protein that plays a crucial role in regulating plant Ca^2+^ balance, resisting abiotic stresses, and transporting heavy metal ions. The peanut *CAX* gene family was identified at the genome-wide level, including analyses of their physicochemical properties, phylogenetic relationships, exon-intron composition, chromosomal localization, gene duplication events, cis-acting elements, transcriptional regulatory network prediction, and expression patterns. The results showed that 10 *AhCAX* genes were identified in the whole genome, distributed on eight chromosomes, and five pairs of gene segment duplication events occurred. These genes encode proteins ranging from 371 to 501 amino acids, with isoelectric points of 5.1 to 8.7. Phylogenetic analysis indicated that *AhCAXs* were divided into two subfamilies, and members of the same subfamily exhibit similar gene structures, conserved motifs, and numbers of transmembrane domains. It was found that hormone-responsive and stress-responsive elements exist in the promoter regions of *AhCAXs*. The expression patterns of *AhCAX* genes showed that *AhCAX1*, *AhCAX4*, *AhCAX7*, *AhCAX8*, and *AhCAX9* were significantly up-regulated under drought or salt stress. Notably, overexpression of the *AhCAX8* gene conferred salt tolerance in transgenic *Arabidopsis* plants by enhancing antioxidant enzyme activity. The results provide a basis for further exploring the function of the peanut *CAX* genes, and provide candidate gene resources for the cultivation of stress-resistant peanut varieties.

## 1 Introduction

Ca^2+^ is an extremely important nutrient element for plant growth and development. It is an essential substance that forms the structure of cell wall and cell membrane, maintaining the stability of the cell membrane and membrane-binding proteins, and reducing or delaying membrane damage (White and Broadley, 2003; Ma et al., 2020; Gifford et al., 2007). CAX (Cation/H^+^ exchanger antiporter), a branch of the Ca^2+^/cation antiporter (CaCA) superfamily, is an important class of transmembrane transporter protein that plays an extremely important role in regulating the change of plant Ca^2+^ content and cation transport (Ueoka-Nakanishi et al., 1999). CAX proteins can be divided into three categories, including type I (plants and some fungal and bacterial CAXs), type II (yeast CAXs), and type III (*Escherichia coli* CAXs). In type I, CAX is further divided into two different subgroups, type IA and IB (Shigaki and Hirschi, 2006; Manohar et al., 2011; Shigaki et al., 2006), implying a functional difference between the two subgroups (Cai and Lytton, 2004). So far, a large number of *CAX* genes have been identified from several plant species, including *Arabidopsis* (Modareszadeh et al., 2021), rice (*Oryza sativa*) (Zou et al., 2021), apple (*Malus domestica*) (Mao et al., 2021), poplar (*Populus*) (He et al., 2022), and *Saccharum* (Su et al., 2021). Using bioinformatics and transgenic methods to identify members of the *CAX* gene family in peanut, studying their chromosome distribution, evolutionary relationships, gene structure, expression under different abiotic stress, and promoter element analysis, is of great significance for studying the function of *CAX* genes in peanut.

CAX is multigene family member localized on the cytoplasmic membrane or the vacuolar membrane (Hirschi et al., 2000). Previous studies found that AtCAX1-AtCAX4 were localized on the vacuole membrane of *Arabidopsis* using immunohybridization and tobacco immunostaining (Gaudet et al., 2011). Furthermore, it has been documented that *Arabidopsis CAX* genes were involved in responses to a variety of abiotic stresses, such as salt, osmotic stress, and heavy metals (Pittman and Hirschi, 2024). The *Arabidopsis* protein kinase SOS2 can regulate plant salt tolerance by interacting with CAX1 (Cheng et al., 2004). In addition, CAX1 has also been shown to function as a negative regulator in *Arabidopsis* anoxia stress, as *cax1–1* and *cax1–2* mutant lines exhibited enhanced tolerance to anoxia stress (Yang et al., 2022). CAX2 and CAX4 have been reported to not only enhance the ability of plants to store heavy metals in the vacuolar zone but also improve the capacity of plants to accumulate Ca^2+^, Cd^2+^, and Mn^2+^ so as to improve the plant tolerance to heavy metals stresses (Manohar et al., 2011; Mei et al., 2009). CAX3 positively regulates Cd tolerance by decreasing reactive oxygen species (ROS) through Ca^2+^ elevation in *Arabidopsis* (Modareszadeh et al., 2021). Furthermore, overexpression of *CAX4* in *Arabidopsis* can enhance the salt tolerance of transgenic plants (Bu et al., 2021). Therefore, the abiotic stresses tolerance of plants can be widely affected by altering the expression of *CAX* genes.

Peanut (*Arachis hypogaea*) is a globally cultivated oilseed and economically significant crop, renowned for its substantial nutritional value and associated social benefits (Zhang et al., 2018). In the peanut cultivation process, abiotic stresses such as extreme temperatures, high salinity, drought, and heavy metals, often harm the metabolism and growth of peanut, leading to quality reduction and yield loss (Qiu et al., 2019; Bhogireddy et al., 2020). For this reason, the widespread use of resistant cultivars able to cope well with a wide range of environmental stresses under field conditions. With the availability of peanut genome sequencing data, a large number of peanut gene families have been identified (Wang et al., 2023; Yu et al., 2023; Wang et al., 2024). However, the role of the peanut *CAX* family genes in regulating abiotic stress response has not been reported. This research analyzed the molecular characteristics of peanut *CAX* family genes and explored the function of *AhCAX8* in regulating response to salt stress. 10 putative CAX members were identified from the peanut genome. The identified CAX members underwent a comprehensive analysis encompassing their phylogenetic relationships, gene structures, promoter regions, chromosomal locations, duplication events, expression patterns, and potential transcriptional regulatory networks, with a particular focus on the functional validation of *AhCAX8* in conferring salt tolerance. Overexpression of the *AhCAX8* gene in *Arabidopsis* conferred plants salt tolerance by enhancing antioxidant enzyme activity. These results provide theoretical basis and genetic resources for studying the function of *CAXs* in peanut abiotic stresses response and breeding resistant varieties.

## 2 Materials and methods

### 2.1 Identification and phylogenetic analysis of the *CaCA* gene family in peanut

The protein sequences of CaCA family members, including six AtCAX, five AtCCX, one AtMHX, and one AtNCL, were obtained from the *Arabidopsis* genome (https://www.arabidopsis.org/). The peanut whole-genome protein sequence was downloaded from the PeanutBase (https://dev.peanutbase.org/). The full-length protein sequences of *Arabidopsis* CaCA members were utilized as queries in a BLASTP program search against the peanut genome database, employing an *E*-value threshold of 1 × 10^-5^. Pfam (Quevillon et al., 2005) and SMART (Letunic and Bork, 2018) were used for the identification of preliminary proteins containing the Na_Ca_ex domain, and proteins lacking this sequence were removed. The amino acid physicochemical properties of peanut CaCA family members such as molecular weight (*Mw*) and isoelectric point (*pI*) were obtained using ProtParam (Wilkins et al., 1999). The subcellular localization of the peanut CaCA family members was predicted through the Plant-mPLoc (Chou and Shen, 2010). MAFFT (version 7.490) (Katoh et al., 2019) was used to compare the peanut AhCAX, protein sequence with the CAX protein sequence of Arabidopsis. The phylogenetic tree was generated by the adjacency algorithm neighbor-joining (NJ) tree in MEGA 11 software, with a bootstrap repeat value of 1–000 times (Tamura et al., 2021).

### 2.2 Analysis of the gene structure and protein motif

GSDS 2.0 (Hu et al., 2015) software was used to analyze the *AhCAX* gene structure by comparing the full-length gene sequence and CDS sequence. MEME (version 5.5.3) (Bailey et al., 2009) was used to analyze and predict the conserved motifs of AhCAX family members in peanut. The protein sequences of AhCAX members were submitted to MEME with the following parameter settings: the number of motifs is set to nine and the length is set to 6-50. The Na_Ca_ex conserved domain of AhCAXs family members was analyzed by SMART (Letunic and Bork, 2018). NovoPro online tool was used to predict the transmembrane region of AhCAXs family members.

### 2.3 Chromosomal localization, duplication event, and collinearity analysis

The position information of *AhCAX* genes was obtained from peanut genome. The distribution of *AhCAX* genes on peanut chromosomes was mapped by MG2C v2.1 (Chao et al., 2021). The duplication events and collinearity of the *AhCAX* genes were analyzed using MCScanX (Wang et al., 2012) and TBtools (version 1.120) (Chen et al., 2020).

### 2.4 Promoter analysis and transcriptional regulatory network prediction

To elucidate the possible molecular mechanisms regulating the *AhCAX* genes, these promoters were analyzed for cis-acting elements. 2kp genomic DNA sequence upstream of the start codon (ATG) of the *AhCAX* gene was obtained from peanut genome database. The *cis*-acting elements in the promoter region were identified using the PlantCARE (Lescot et al., 2002).

In order to predict the upstream transcription factors of *AhCAX* genes, the 2kb promoter sequence of *AhCAX* genes were submitted to PlantRegMap database. The transcription factors that regulate *AhCAX* genes were then predicted using Binding Site Prediction tool with *p* value of ≤ 10^−5^ (Tian et al., 2020).

### 2.5 Growth and stress treatments of peanut plants

To analyze the expression of *AhCAX* genes, peanut seeds were germinated on MS medium at 25 °C for two weeks. The resulting seedlings were treated with 20% PEG 6000 (drought) or 200 mM NaCl (salt). Leaves were harvested at 0, 3, and 6 h, immediately frozen in liquid nitrogen, and stored at -80 °C. Total RNA was extracted for subsequent qRT−PCR analysis. Each treatment comprised three biological replicates.

### 2.6 Expression profiles and qRT-PCR of *AhCAX* genes

Gene expression data of peanut different plant tissues, including leaf 1, leaf 2, leaf 3, perianth, veg shoot, repr shoot, stamen, root, nodule, and pistil, was obtained from Peanut Base (accession: PRJNA291488). The expression data of peanut *AhCAX* genes under drought and salt stress were downloaded from NCBI (Zhang et al., 2020; Zhao et al., 2018). Using TBtools (Chen et al., 2020) to draw the heat map of *AhCAX* gene expression pattern in different tissues and under drought and salt stress.

For qRT-PCR, first-strand cDNA was synthesized using the TransGen Biotech cDNA Synthesis Kit. Quantitative real-time PCR (qRT-PCR) was subsequently carried out on an ABI 7900HT system (Applied Biosystems) with SYBR Green Master Mix (TaKaRa, Japan). Each 20 μL reaction contained a pair of gene-specific primers. Transcript levels were normalized to the peanut *Actin11* gene (Wang et al., 2023), and relative expression was calculated using the 2^-ΔΔCT^ method (Livak and Schmittgen, 2001). All primer sequences are listed in **Supplementary Table S1**.

### 2.7 Overexpression analysis

The full-length CDS sequence of *AhCAX8* gene was inserted into the expression vector pCHF3 to obtain the fusion construct *pCHF3-35S::AhCAX8*. The construct was then transformed into *Arabidopsis* by floral dip method (Zhang et al., 2006). The PCR and kanamycin were used to identified the homozygous lines. WT and two *AhCAX8-OE* lines (*OE-7* and *OE-9*) that grew normally for seven days were transferred to 1/2 MS medium containing 75 and 100 mM NaCl, respectively. The medium was placed in a light incubator (23 °C, continuous light). Primary root length was measured after one week of growth.

### 2.8 Determination of Malondialdehyde content and enzyme activity

The levels of malondialdehyde (MDA) and the enzymatic activities of superoxide dismutase (SOD), peroxidase (POD), and catalase (CAT) were determined in wild−type and *AhCAX8*-*OE* plants using corresponding commercial kits (Solarbio, Beijing, China). The experiments were conducted with three biological replicates.

### 2.9 Statistical analysis

The GraphPad Prism 8 was used to analyze significant differences (*P* < 0.05). The values were determined from three biological replicates. Different lowercase letters (a, b, and c) above the bar represent statistically significant differences between columns.

## 3 Results

### 3.1 Identification of *AhCAX* genes

A total of 10 AhCAX members were identified from peanut genomic database using BLAST method. The 10 *AhCAX* genes were named from *AhCAX1* to *AhCAX10* based on where they are located on the chromosomes. The physiochemical features of AhCAXs such as the number of amino acids (aa), molecular weight (*Mw*), and isoelectric point (*pI*) were determined. Results showed that the length of proteins ranged from 371 (AhCAX3 and AhCAX8) to 501 (AhCAX7) aa. The *Mw* of these AhCAXs ranged from 40260.21 Da (AhCAX3) to 55144.08 Da (AhCAX7), while their *pI* varied from 5.1 (AhCAX2) to 8.7 (AhCAX3) (**Supplementary Table S2**).

### 3.2 Phylogenetic relationship and gene structure of AhCAXs

**Figure 1:**
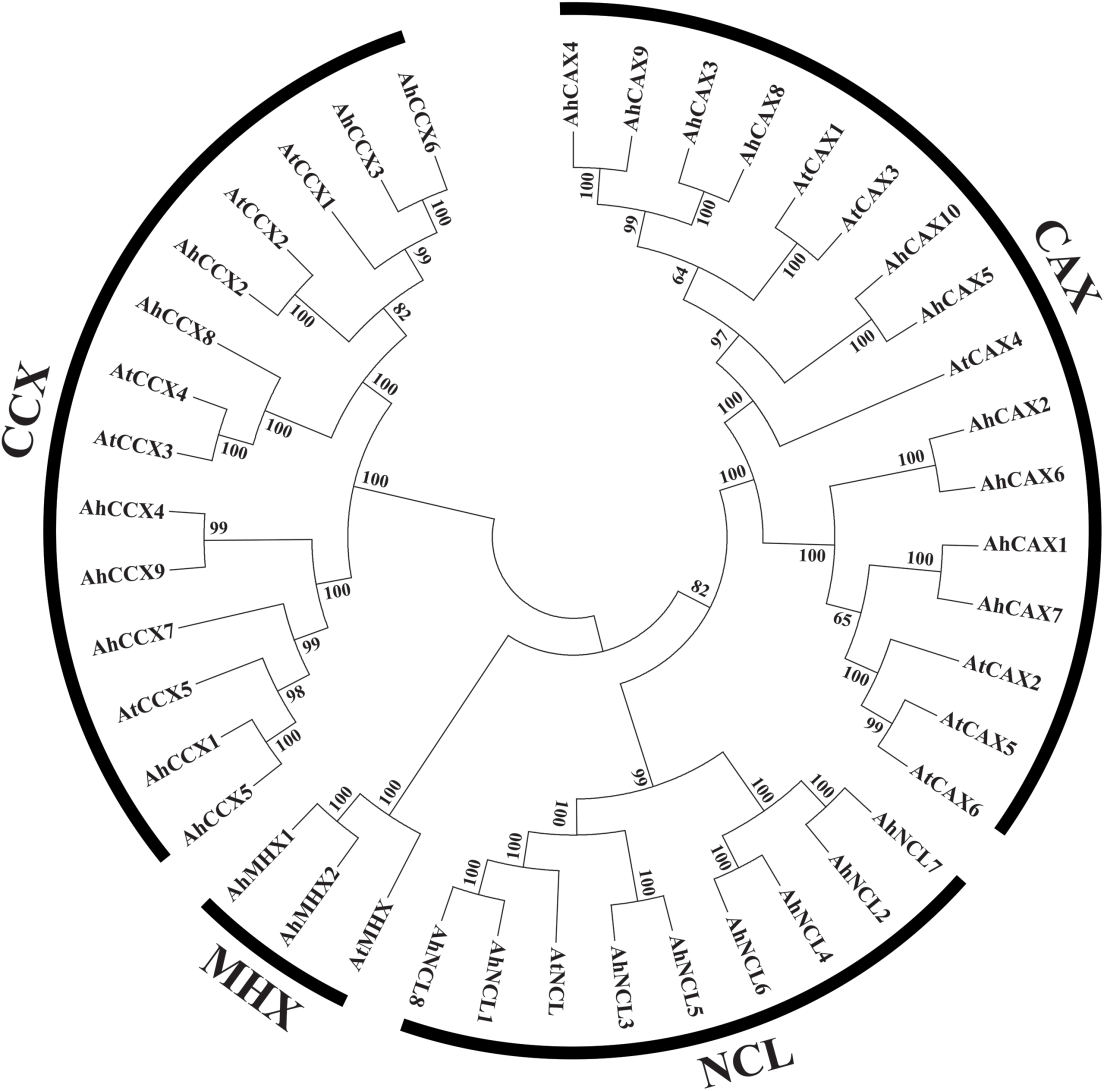
The phylogenetic relationship of CAXs from peanut and *Arabidopsis*.

**Figure 2:**
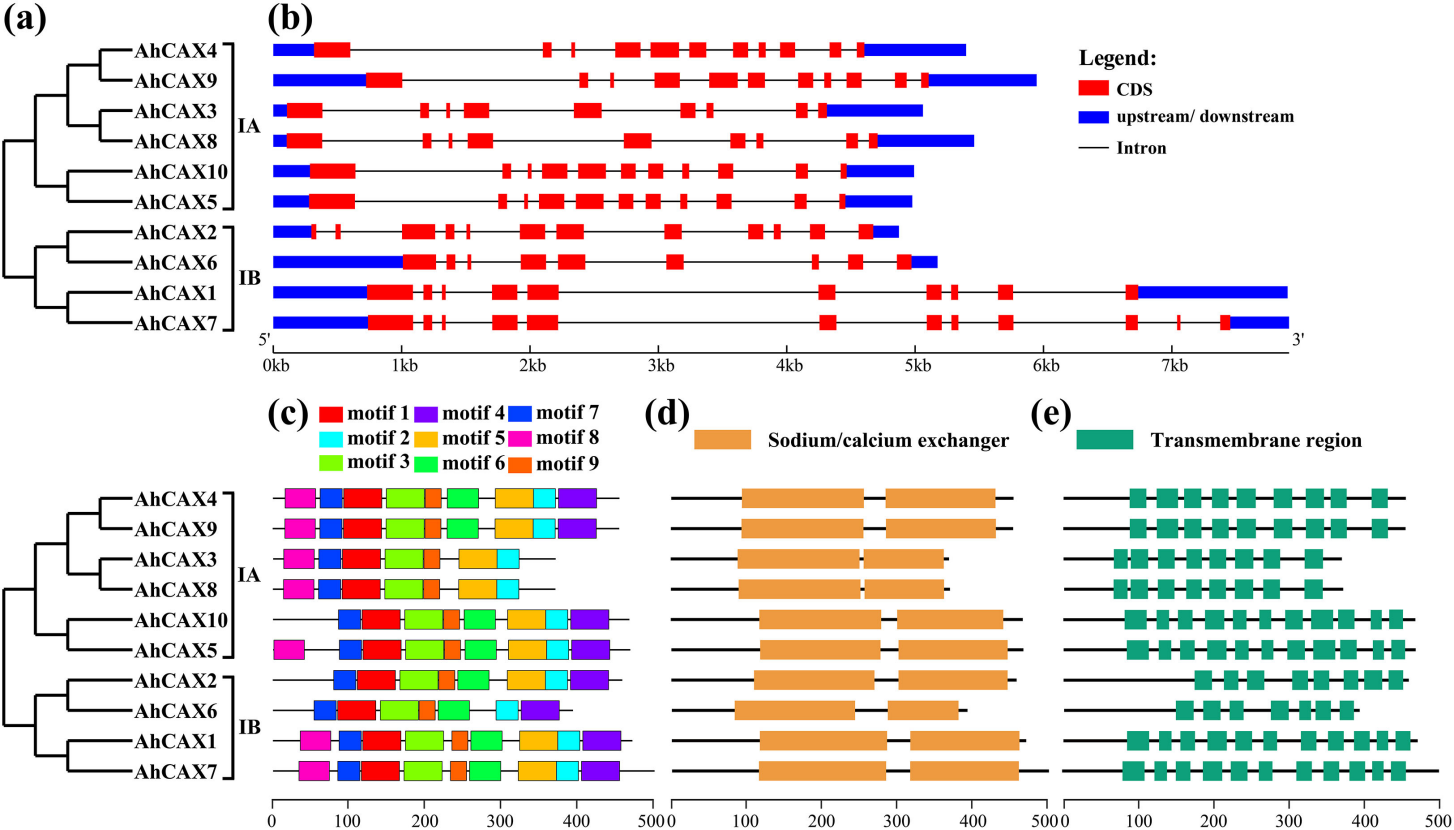
Gene structure and conserved motif analysis of AhCAX members. **(a)** Phylogenetic tree of 10 AhCAX members. **(b)** Exon-intron structure. Yellow boxes represent exons, black lines represent introns, and blue boxes represent untranslated regions (UTRs). **(c)** Distribution of conserved motifs. Nine putative motifs are represented by different colored boxes. **(d)** Conserved domain analysis showing the Na_Ca_ex domain. **(e)** Predicted transmembrane domains.

To investigate the phylogenetic relationship of the *CAX* gene family members, a NJ tree was constructed among CAX protein sequences from *Arabidopsis* and peanut. Phylogenetic analysis showed that the 10 *AhCAXs* were classified into two subfamilies (IA and IB) together with their *Arabidopsis* homologs (**Figure 1**). Six *AhCAXs* were grouped into the subfamily IA, while four *AhCAXs* belonged to the subfamily IB (**Figure 2a**), suggesting that some *AhCAX* genes might have undergone whole genome duplication during peanut’s evolutionary process.

The examination of the gene structure showed that most *AhCAXs* in same subfamily had similar gene structures (**Figure 2b**). A total of nine motifs were identified in AhCAX proteins. It was found that the protein motif composition of 10 AhCAX members was generally consistent (**Figure 2c**). The results suggest that the AhCAX members of the same subfamily might have similar functions.

### 3.3 Chromosomal distribution, duplication events, and syntenic analysis

**Figure 3:**
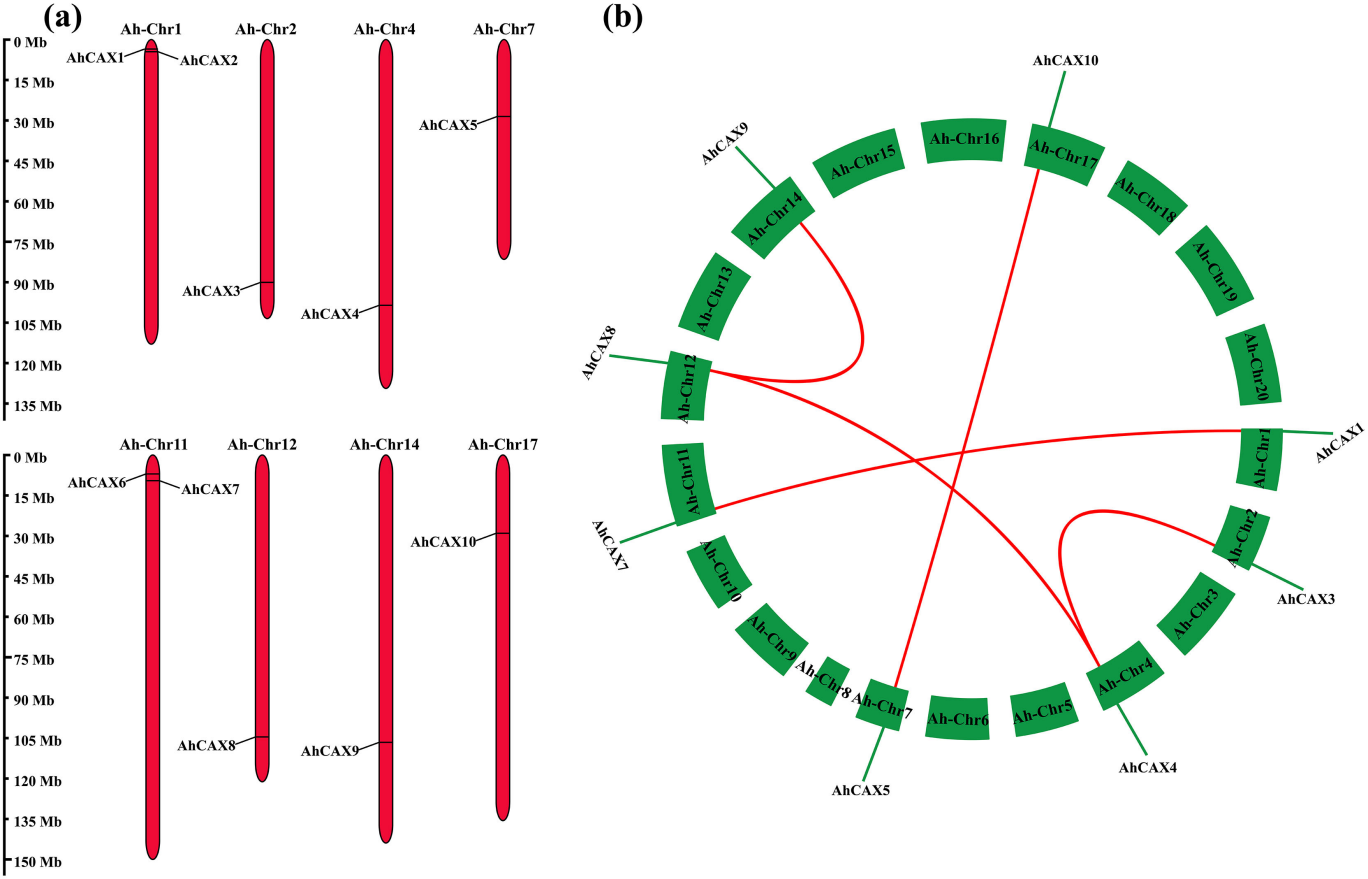
Chromosomal distribution and duplication events analysis of peanut *CAX* genes. **(a)** Eight chromosomes contained a total of 10 *AhCAX* genes that were mapped. **(b)** Five segmental duplication pairs of *AhCAX* genes were linked by the red lines.

The chromosome distribution map of the *AhCAX* genes was generated. The results indicated that 10 *AhCAX* genes were distributed on eight peanut chromosomes (**Figure 3a**). Ah-Chr1 and Ah-Chr11 possessed the maximum number of two *AhCAX* genes, while one *AhCAX* gene was found in Ah-Chr2, Ah-Chr4, Ah-Chr7, Ah-Chr12, Ah-Chr14, and Ah-Chr17. Five segmental duplication gene pairs were found in eight *AhCAX* genes (**Figure 3b**; **Supplementary Table S3**). The results suggest that specific *AhCAX* genes may have arisen through gene duplication, with segmental duplication events possibly playing a crucial role in the evolutionary development of *AhCAX* genes. Furthermore, analysis of the five gene pairs revealed a *Ka*/*Ks* ratio of less than 1, implying that these *AhCAX* genes have likely been subject to purifying selection during their evolutionary history (**Supplementary Table S3**).

**Figure 4:**
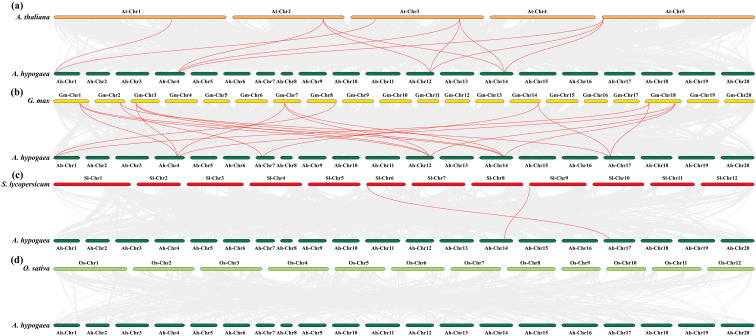
Syntenic analysis of *CAX* genes in peanut, *Arabidopsis*, soybean, tomato, and rice. **(a)** *Arabidopsis* and peanut. **(b)** Soybean and peanut. **(c)** Tomato and peanut. **(d)** Rice and peanut. The red lines show the syntenic *CAX* gene pairs.

In order to enhance comprehension of the phylogenetic relationships within the peanut *CAX* gene family, a syntenic analysis was conducted comparing the CAX genes of peanut with those of four other plant species: *Arabidopsis*, soybean, tomato, and rice (**Figure 4**). The results revealed that six, four, two, and zero *AhCAX* genes were synchronized with *CAX* genes in soybean, *Arabidopsis*, tomato, and rice, respectively. There were 19, 11, 2, and 0 collinear pairs between peanut and the other four plant species (soybean, tomato, *Arabidopsis*, and rice), indicating a close genetic relationship between peanut *CAX* genes and soybean *CAX* genes (**Supplementary Table S4**).

### 3.4 Cis-regulatory elements analysis and regulatory network prediction

**Figure 5:**
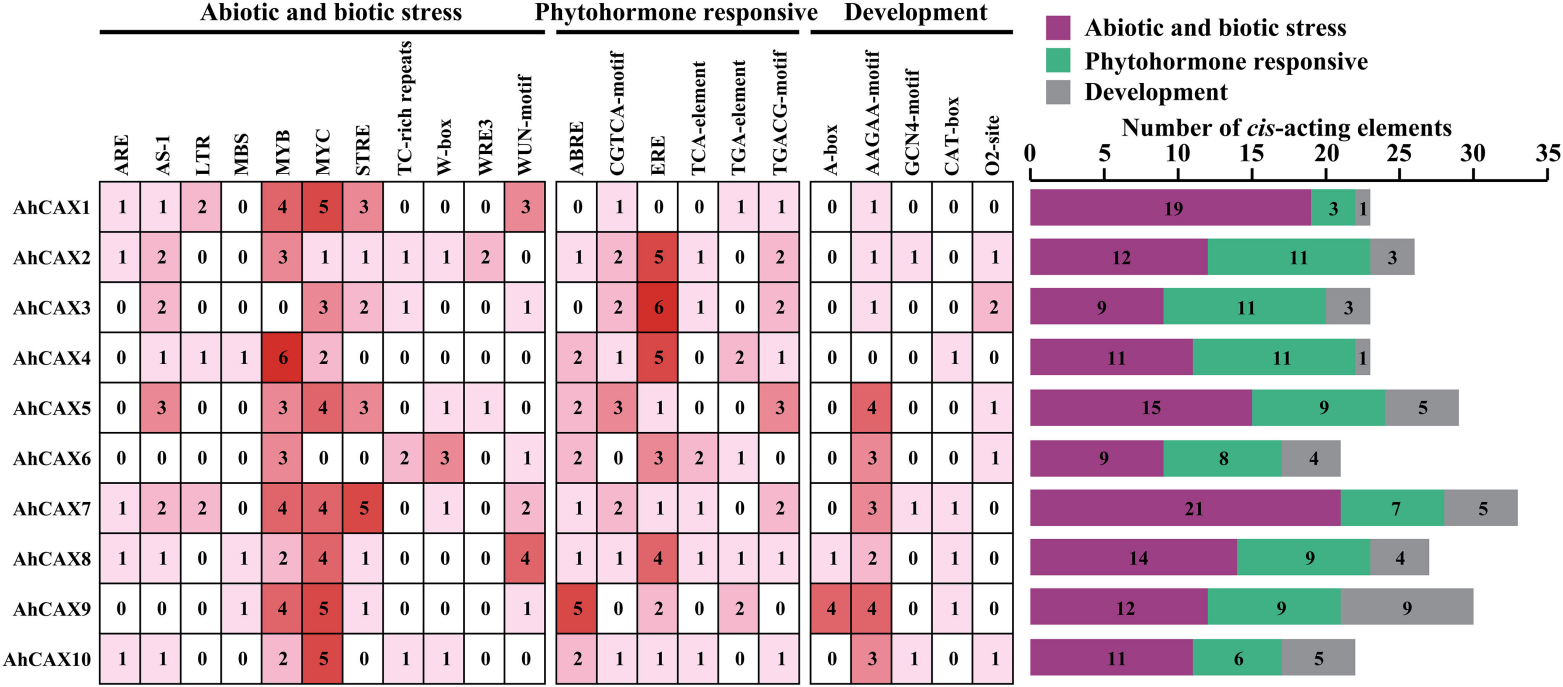
*Cis*-regulatory elements (CREs) analysis.

To explore the potential functions of peanut *CAX* genes, *cis*-regulatory elements (CREs) from 2 kp of these gene promoters were analyzed using the PlantCARE tool. 22 types of CREs related to abiotic/biotic stress response, phytohormone response, and development were identified (**Figure 5**). For example, ABA response elements (ABREs) were found in the promoters of eight *AhCAX* genes, while TCA-elements were found in the promoters of six *AhCAX* genes. Abiotic stress response elements including STRE, TC-rich repeats, W-box, and WUN-motif were found in the promoters of seven, four, five, and six *AhCAX* genes, respectively. Results showed that *AhCAX* genes might respond to abiotic stresses in peanut.

**Figure 6:**
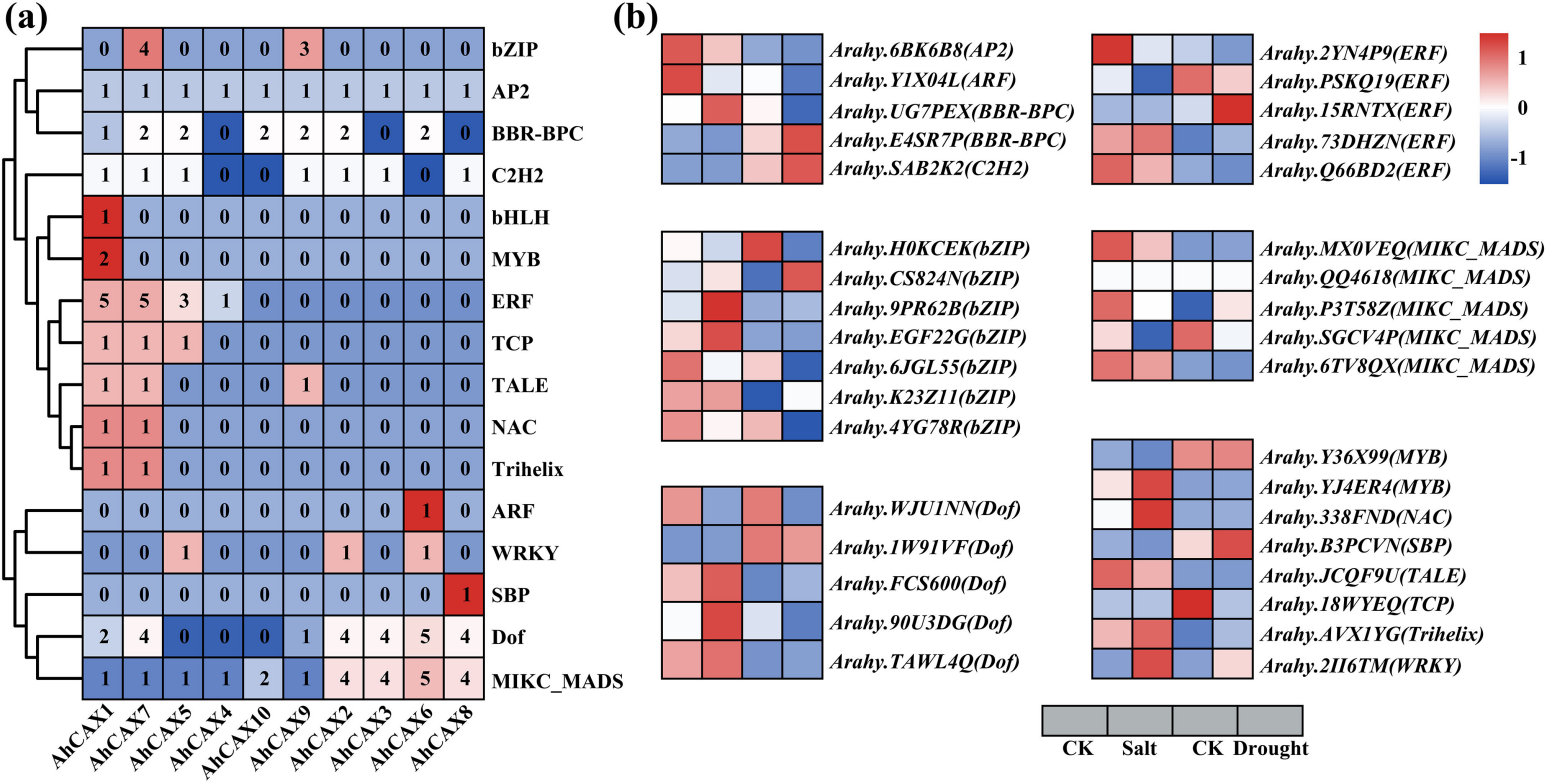
Regulatory network prediction. **(a)** The number of potential transcription factors (TFs) that bind the *AhCAX* gene. **(b)** The expression patterns of these TF coding genes under drought and salt treatments. The FPKM values of each TF were normalized and clustered using TBtools.

To investigate the regulatory characteristics of *AhCAX* genes, the PlantRegMap was used to predict potential transcription factors (TFs). The results showed that transcription of the *AhCAX* genes is mainly regulated by 16 types of TFs (**Figure 6a**). Almost all *AhCAX* genes might be regulated by AP2, BBR-BPC, C2H2, Dof, and MIKC_MADS TFs. Furthermore, *AhCAX7* was regulated by the most TFs (22), whereas *AhCAX4* was regulated by the least (three). In addition, the expression patterns of these TFs encoding genes under drought and salt stress were analyzed using RNA-seq data (**Figure 6b**).

### 3.5 Expression profiles of *AhCAXs* in different tissues and response to salt stress

**Figure 7:**
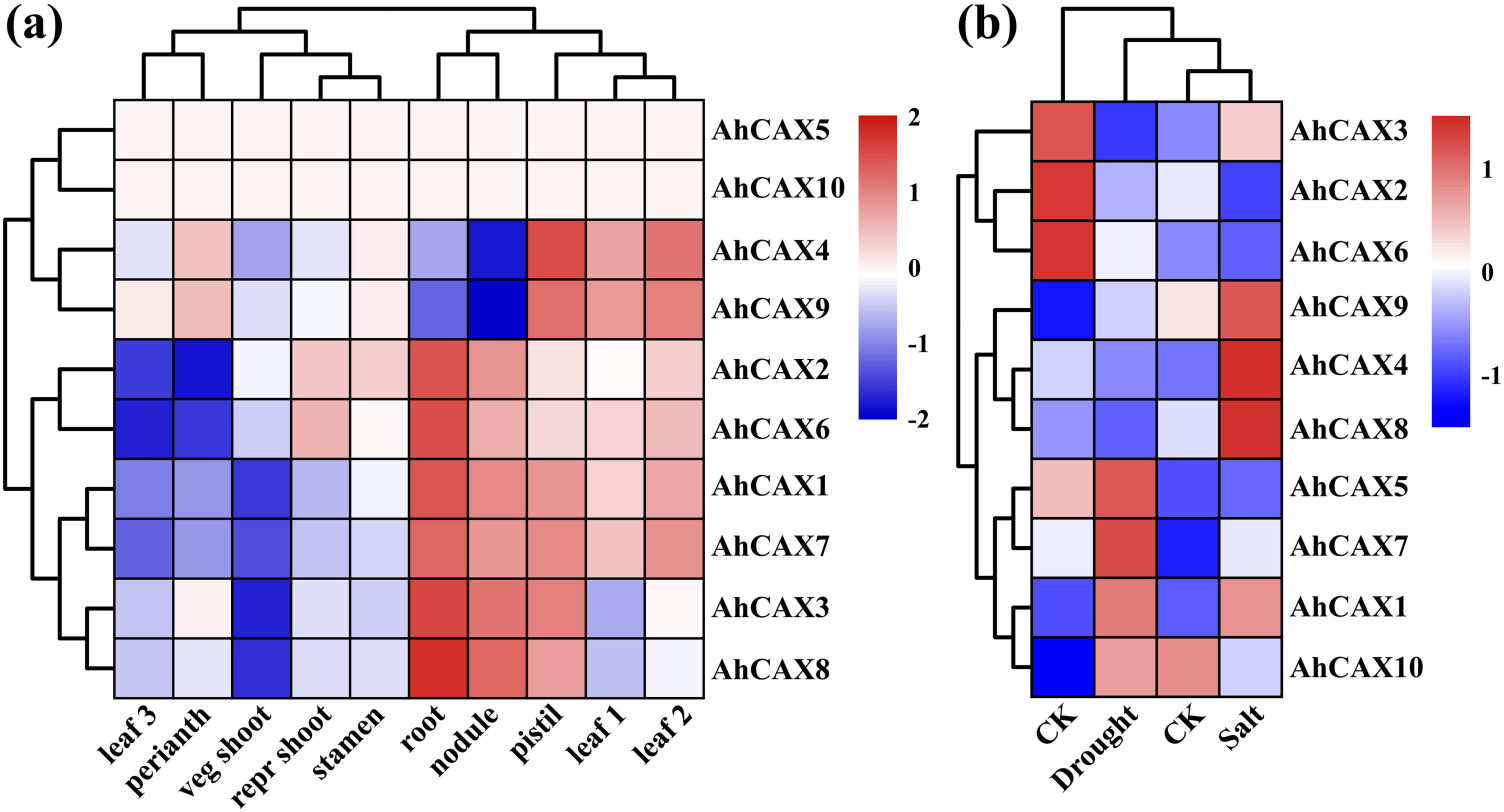
Expression profiles of *AhCAX* genes in different tissues and response to abiotic stress. **(a)** Transcriptome-based expression profiling of *AhCAX* genes from 10 peanut tissues consisted of leaf, shoot, root, nodule, perianth, stamen, and pistil. **(b)** Transcriptome-based expression profiling of *AhCAX* genes under drought and salt treatments. The FPKM values of each *AhCAX* were normalized and clustered using TBtools.

To assess the expression patterns of *AhCAX* genes, the transcriptome data of eight peanut tissues (leaf, veg shoot, repr shoot, root, nodule, perianth, stamen, and pistil) were normalized. Results showed that several *AhCAX* genes, including *AhCAX1*, *AhCAX2*, *AhCAX3*, *AhCAX6*, *AhCAX7*, and *AhCAX8*, were highly expressed in root tissue (**Figure 7a**). Considering the root tissue was an important organ in plants in response to abiotic stresses, it is plausible to suggest that these *AhCAX* genes could play a role in the response to abiotic stresses in peanut.

To further explore the response of *AhCAX* genes to abiotic stresses, the RNA-seq data in root tissues under drought and salt stress was analyzed. Analysis of the expression patterns indicated that three *AhCAX* genes, including *AhCAX1*, *AhCAX7*, and *AhCAX9*, were up-regulated under drought and salt stress. The expression of *AhCAX5* and *AhCAX10* was up-regulated under drought stress, while down-regulated under salt stress. However, *AhCAX3*, *AhCAX4*, and *AhCAX8* exhibited up-regulated expression patterns under salt stress and down-regulated expression patterns under drought stress (**Figure 7b**). The diverse expression patterns observed in *AhCAX* genes indicate their distinct roles in drought or salt stress-response pathways.

### 3.6 Overexpression of *AhCAX8* enhanced salt tolerance in *Arabidopsis*

**Figure 8:**
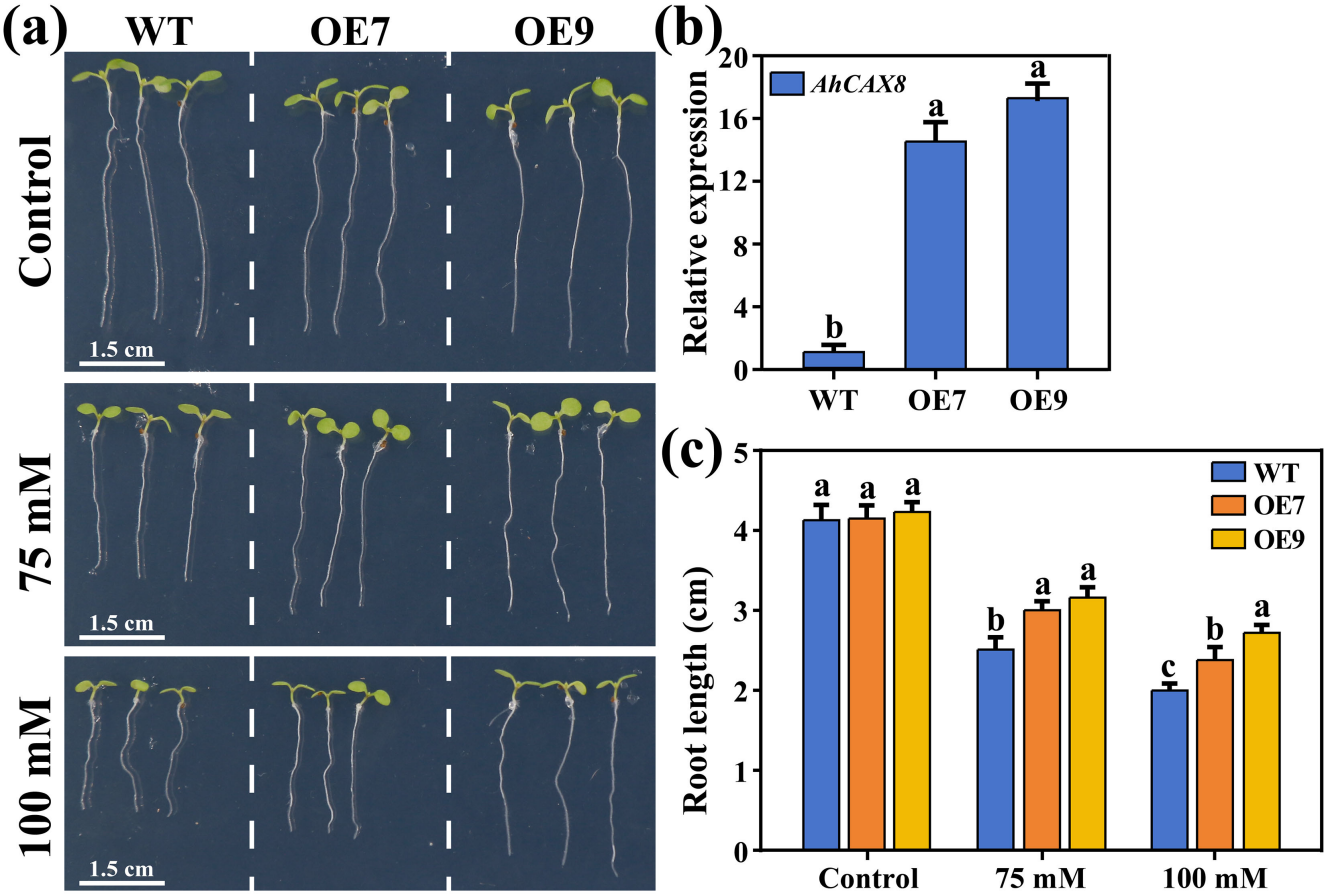
AhCAX8 was well confirmed to positively regulate salt stress in transgenic *Arabidopsis*. **(a)** Phenotype changes of the WT and two *AhCAX*-*OE* lines under the control and salt stress (75 mM and 100 mM NaCl). **(b)** The expression of *AhCAX8* in the WT and two *AhCAX8*-*OE* lines (*OE7* and *OE9*). **(c)** The primary root lengths under the control and salt stress. WT, wild-type. Different lowercase letters (a–c) above the bar represent statistically significant differences between columns (*P* < 0.05).

To further explore the potential function of *AhCAX8* in salt stress response, transgenic lines overexpressing *AhCAX8* were generated in *Arabidopsis* plants (*AhCAX8-OE*). Two lines, *AhCAX8*-*OE7* and *AhCAX8*-*OE9*, were chosen for further analysis due to the level of *AhCAX8* expression in the *AhCAX8*-*OE* lines (**Figure 8b**). Compared with WT, the *OE7* and *OE9* lines exhibited a phenotype of longer roots under salt treatments (**Figures 8a, c**), suggesting that overexpression of *AhCAX8* could enhance salt tolerance in transgenic *Arabidopsis*.

**Figure 9:**
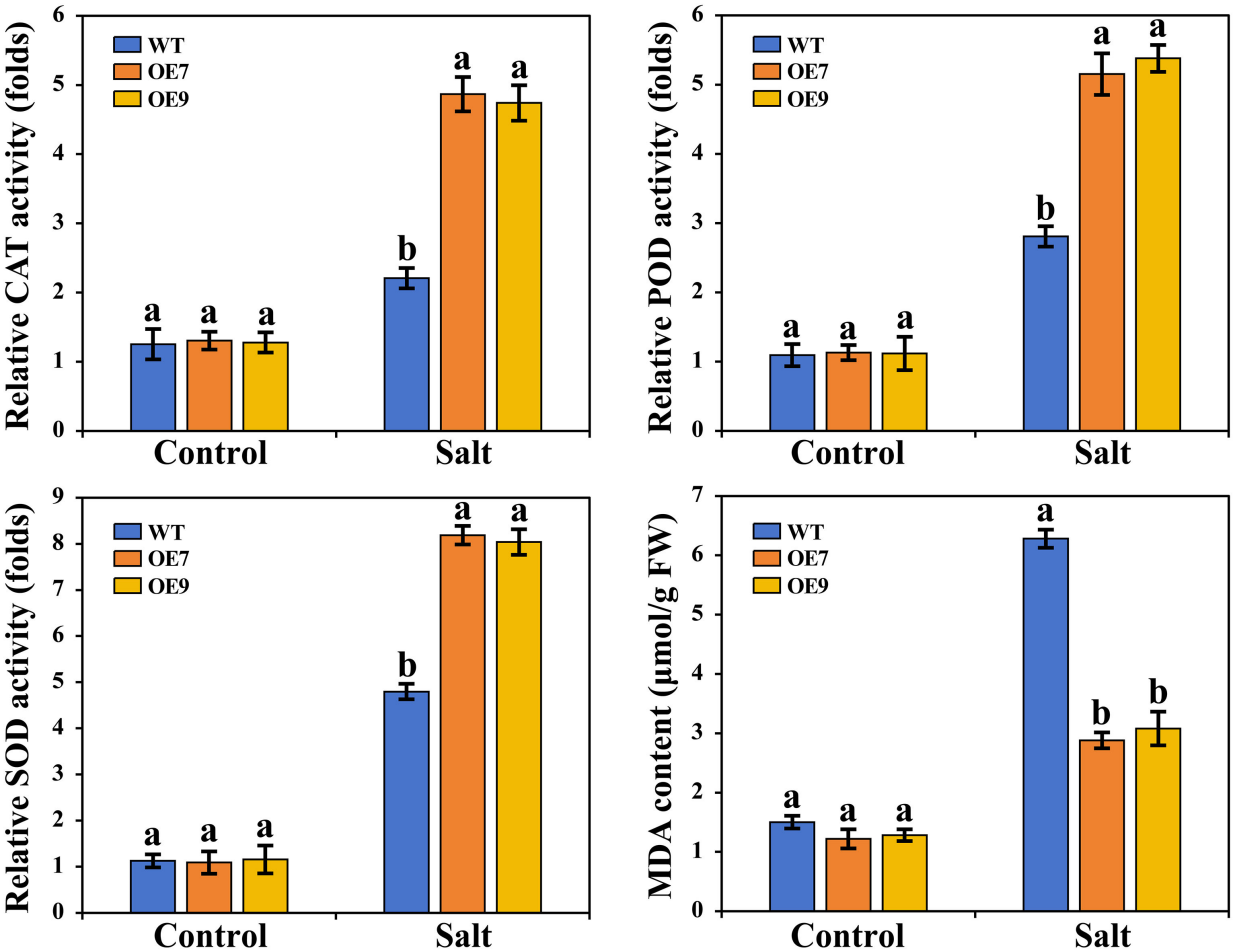
Changes of antioxidant enzyme activities and MDA content in *AhCAX8* overexpressing and wild-type peanut after salt stress. MDA, malonicdialdehyde; SOD, superoxide; POD, peroxidase; CAT, catalase. WT, wild-type. Different lowercase letters (a–c) above the bar represent statistically significant differences between columns (*P* < 0.05).

When subjected to salt stress, the *OE7* and *OE9* lines showed stronger antioxidant enzyme activity and significantly lower MDA content compared to those of the WT (**Figure 9**). The results indicated that *AhCAX8*-*OE* lines might have enhanced salt stress tolerance by enhancing antioxidant enzyme activity.

## 4 Discussion

CAX is a Ca^2+^/H^+^ antiporter and is a transmembrane protein of a class of divalent ion transporters (Pittman and Hirschi, 2024; Shigaki et al., 2010). CAX is widely found in bacteria, fungi, plants, and lower vertebrates (Cai and Lytton, 2004). Currently, the *CAX* gene families have been identified in multiple species. For example, there are six CAX family members in *Arabidopsis* (Emery et al., 2012), six CAX family members in rice (Zou et al., 2021), 11 CAX family members in apple (Mao et al., 2021), and 14 CAX family members in wheat (*Triticum aestivum*) (Taneja et al., 2016). However, a systematic study of peanut *CAX* gene family has not been reported. In this study, we identified 10 *CAX* gene members in the whole peanut genome, which is more than number found in the diploid plants *Arabidopsis* and rice. This might be because the peanut is allotetraploid, resulting in a greater number of homologous genes. In addition, these *AhCAX* genes encode proteins ranging from 371 to 501 amino acids, with isoelectric points of 5.1 to 8.7. Apple *CAX* gene families also exhibited similar physiological characteristics (Mao et al., 2021). *AhCAX3*, *AhCAX4*, *AhCAX5*, *AhCAX8*, *AhCAX9*, and *AhCAX10* belong to subfamily IA, while *AhCAX1*, *AhCAX2*, *AhCAX6*, and *AhCAX7* belong to subfamily IB (**Figure 1**), suggesting a possible functional divergence of peanut *CAX* genes between subfamily IA and subfamily IB.

Previous studies showed that protein structure is closely related to its function. In this study, CAX members within the same subfamily all exhibited similar structures, and the gene structure and conserved motifs of these members were generally consistent with the phylogenetic analysis, and the number of transmembrane domains was also similar (**Figure 2**). Similar results were found in other species, such as *Arabidopsis*, rice (Zou et al., 2021), and apple (Mao et al., 2021), suggesting that the biological function of the CAX family is relatively conserved during evolution. Gene duplication events are an important mechanism for gene family expansion (Vision et al., 2000). Gene duplication includes segmental duplication and tandem duplication, both of which are significant drivers of biological evolution. Among these gene duplication events, segmental duplication is one of the major contributors to the expansion of many gene families (Cannon et al., 2004). In this study, five pairs of duplication events occurred in peanut *CAX* gene family, all of which were segmental duplication events (**Figure 3b**; **Supplementary Table S3**). Similarly, apple *CAX* gene families also exhibited segmental duplication events (Mao et al., 2021). The results indicated that segmental duplication events may be the main driving force for the expansion of *CAX* gene family members during evolution. In addition, this study found that all homologous genes are in the same evolutionary clade (**Figures 1**, **3b**), indicating the high retention rate of homology in peanut. Our phylogenetic analysis clearly divided AhCAXs into two subfamilies, IA and IB (**Figure 1**). Interestingly, members of subfamily IA (like *AhCAX1* and *AhCAX7*) showed broader expression across various tissues and responded to both drought and salt stress, while subfamily IB members (like *AhCAX4*, *AhCAX8*, and *AhCAX9*) were more specifically induced by salt stress (**Figure 7b**). This suggests a potential functional divergence, where IA members may play a more general role in development and stress homeostasis, whereas IB members might be specialized for ionic stress responses, particularly salinity. This hypothesis is supported by the distinct cis-element compositions in their promoters (**Figure 5**) and warrants further investigation through targeted functional studies of individual members from each subfamily.

All promoters in the peanut *CAX* gene family members have *cis*-acting elements in response to abiotic stress, such as LTR responding to low temperature, ARE associated with low and oxygen stress, MBS associated with drought stress, and TC-rich in response to defense and stress (**Figure 5**), indicating that *AhCAXs* might play an important role in response to abiotic stresses. Transcription factors can regulate gene expression at the transcriptional level by binding to *cis*-acting elements in downstream gene promoters. Some studies have found that cis-acting elements such as MYB, W-box, MBS, and LTR play important roles in regulating plant *CAX* genes in response to environmental stresses (Yang et al., 2023). In this study, prediction analysis of transcriptional regulation indicated that the peanut *CAX* gene is regulated by several transcription factors, such as Dof, bZIP, ERF, and MIKC_MADS (**Figure 6a**). Notably, the expression of genes coding for these transcription factors is also induced by drought or salt stress (**Figure 6b**). These results indicated that *AhCAX* genes might be involved in peanut response to abiotic stresses by regulation of transcription factors. Moreover, the *CAX* gene family showed different expression patterns in a variety of tissues (**Figure 7a**). The *AhCAX4* and *AhCAX9* genes were specifically expressed in leaves, pistil, and perianth, while the *AhCAX3* and *AhCAX8* genes were expressed in roots, nodule, and pistil. In addition, the *AhCAX1*, *AhCAX2*, *AhCAX6*, and *AhCAX7* genes were expressed in almost all of the tissues examined. Furthermore, the promoters of these *CAX* genes contain multiple cis-acting elements related with growth and development (**Figure 5**). The results showed that *AhCAX* genes play important roles in different tissues and developmental stages of peanut.

The CAX proteins of several plant species have been shown to have important roles in response to abiotic stresses (Pittman and Hirschi, 2016). *Arabidopsis cax3-1*, *cax3-2*, and *cax1*/*cax3* mutants showed significant sensitivity to salt stress. Furthermore, *atcax1* and *atcax4* mutants showed increased sensitivity to salt stress with increasing NaCl concentration (Zhao et al., 2008). Tolerance to salt stress in lines overexpressing *AtCAX1* or *AtCAX4* significantly enhanced (Bu et al., 2021). Under salt stress, the expression of *AhCAX3*, *AhCAX4*, *AhCAX8*, and *AhCAX9* was significant up-regulation (**Figure 7b**). In addition, these four *AhCAX* genes were located in the same evolutionary clade together with *AtCAX1*, *AtCAX3*, and *AtCAX4* (**Figure 1**). The *Arabidopsis cax1* mutants (*atcax1–3* and *atcax1-4*) showed increased tolerance at low temperature than the wild type, suggesting that *AtCAX*1 plays a negative regulatory role at low temperature (Catala et al., 2003). This result is consistent with the fact that *AtCAX1* overexpression of tobacco showed stronger cold sensitivity (Hirschi, 1999). Promoter analysis showed that *AhCAX1*, *AhCAX4*, and *AhCAX7* had low temperature stress response elements (**Figure 5**), indicating that these genes might play important role in regulation during cold stress. It was found that the expression of *GmCAX1* was increased under drought stress (Luo et al., 2005). *AhCAX1*, *AhCAX5*, *AhCAX7*, and *AhCAX10* were significantly up-regulated under drought stress (**Figure 7b**), indicating that these genes may be involved in the regulation of peanut drought resistance.

In this study, the phylogenetic analysis showed that *AhCAX8* and *AtCAX1*/*3*/*4* were diversified into the same clade and have high homology (**Figure 1**). In addition, salt stress can significantly induce the expression of *AhCAX8* (**Figure 7b**). Therefore, *AhCAX8* was selected for the further functional analysis based on these results. Transgenic analysis demonstrated that the transgenic *Arabidopsis* lines overexpressing *AhCAX8* gene displayed increased root length when exposed to salt stress, as compared to the wild-type plants (**Figure 8**). When plants are subjected to abiotic stress, either individually or in combination, they generate an excess of ROS, resulting in oxidative stress and disrupted redox homeostasis. This process ultimately culminates in the formation of malondialdehyde (MDA), the end product of lipid peroxidation in plant cell membranes (Hasanuzzaman et al., 2020). Transgenic *Arabidopsis* lines overexpressing *AhCAX8* gene under salt stress, MDA content was decreased, while activities of SOD, POD, and CAT were increased (**Figure 9**), suggesting that overexpression lines increased the ROS-scavenging capacity.

From a practical perspective, the enhanced salt tolerance conferred by *AhCAX8* overexpression in Arabidopsis highlights its potential as a promising candidate gene for molecular breeding of stress-resilient peanut cultivars. Future work could involve overexpressing *AhCAX8* in peanut itself using a root-specific or stress-inducible promoter to minimize potential pleiotropic effects. Additionally, exploring natural allelic variations of *AhCAX8* in different peanut germplasm accessions could provide valuable markers for marker-assisted selection in breeding programs aimed at improving salt tolerance.

## 5 Conclusions

In conclusion, 10 *AhCAX* genes were systematically identified from the peanut genome and were analyzed comprehensively. Several *AhCAX* genes, including *AhCAX1*, *AhCAX4*, *AhCAX7*, *AhCAX8*, and *AhCAX9*, exhibited significant changes in expression levels when exposed to drought or salt stress in peanut. Notably, *Arabidopsis* lines overexpressing *AhCAX8* displayed longer root length and stronger antioxidant enzyme activity compared to WT, indicating *AhCAX8* plays an important role in regulating plant abiotic stress tolerance. Together, these findings can help us better understand peanut responses to abiotic stress and aid in the effective strategies aimed for improving abiotic stress tolerance in peanut crop. In future study, we will explore the regulatory networks that control the expression of *AhCAX* genes.

## Data Availability

The original contributions presented in the study are included in the article/**Supplementary Material**. Further inquiries can be directed to the corresponding author.

## Supplementary material

Supplementary Figure 1 The expression patterns of *AhCAX* genes under drought and salt conditions. **(A)** The expression pattern of selected *AhCAX* genes in response to drought stress treatments, which was calculated as folds relative to the untreated control. **(B)** The expression pattern of selected AhHsf genes in response to salt stress treatments, which was calculated as folds relative to the control. Different lowercase letters (a–c) above the bar represent statistically significant differences between columns (*P* < 0.05).

Supplementary Table 1 The primers used in this study.

Supplementary Table 2 The detail information of identified cultivated peanut CAX family members.

Supplementary Table 3 The detail information of segmental duplication gene pairs.

Supplementary Table 4 The syntenic pairs between peanut and other four plant species.
