# Supplementary figures and images for: Identification of the cation/H+ exchanger genes in peanut and functional analysis of AhCAX8 in response to salt stress
Source: Front Plant Sci. 2026 Jul 16;17:1900784. doi: 10.3389/fpls.2026.1900784 (PMC13422565; doi:10.3389/fpls.2026.1900784)

**(a)**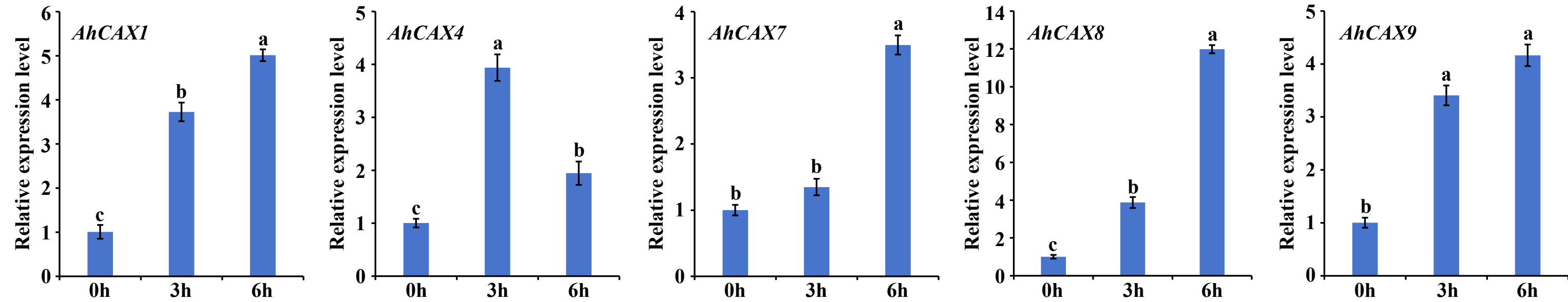**Drought stress****(b)**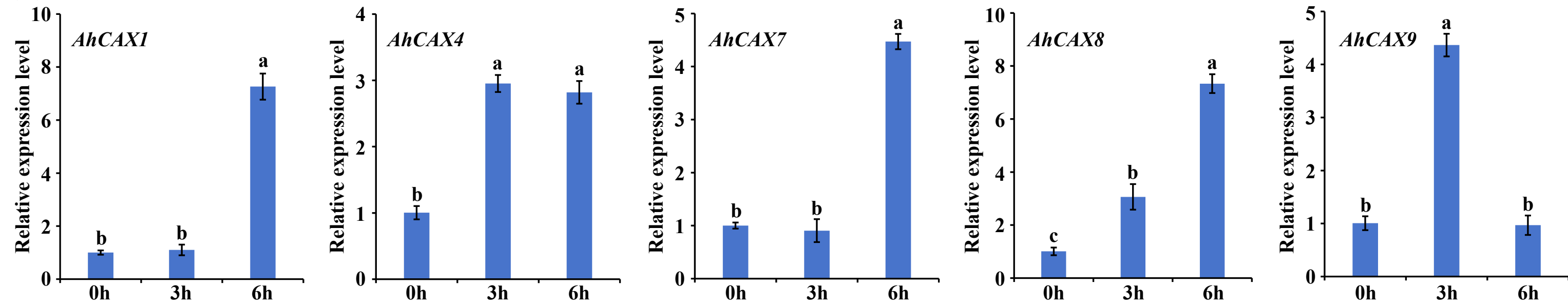**Salt stress**

Supplement: Supplementary Figure 1 — The expression patterns of AhCAX genes under drought and salt conditions. (A) The expression pattern of selected AhCAX genes in response to drought stress treatments, which was calculated as folds relative to the untreated control. (B) The expression pattern of selected AhHsf genes in response to salt stress treatments, which was calculated as folds relative to the control. Different lowercase letters (a–c) above the bar represent statistically significant differences between columns (P < 0.05). [file DataSheet1.zip › Supplementary Materials/Figure S1.pdf]
